# The impact of liver abscess formation on prognosis of patients with malignant liver tumors after transarterial chemoembolization

**DOI:** 10.3389/fonc.2023.1256012

**Published:** 2023-11-07

**Authors:** Yunan Wang, Zhihui Chang, Jiahe Zheng, Zhaoyu Liu, Jun Zhang

**Affiliations:** Department of Radiology, Shengjing Hospital of China Medical University, Shenyang, China

**Keywords:** transarterial chemoembolization, percutaneous drainage, liver abscess, malignant liver tumors, prognosis

## Abstract

**Purpose:**

Liver abscess is a rare and serious complication after transarterial chemoembolization (TACE) for liver cancer; however, its impact on the prognosis is unclear. This retrospective study examined the outcomes of patients with liver abscess formation following TACE for malignant liver tumors to elucidate the impact of liver abscess formation on the prognosis of these patients.

**Methods:**

From January 2017 to January 2022, 1,387 patients with malignant tumors underwent 3,341 sessions of TACE at our hospital. Clinical characteristics of patients at baseline and follow-up were examined, including treatment and outcome of liver abscess, tumor response to the TACE leading to liver abscess, and overall survival time.

**Results:**

Of 1,387 patients, 15 (1.1%) patients with liver abscess complications after TACE resulted in a total of 16 (0.5%) cases of liver abscess after 3,341 TACE sessions (including one patient with two events). After antibiotic or percutaneous catheter drainage (PCD) treatment, all the infections associated with liver abscesses were controlled. In the PCD group, eight patients died before drainage tube removal, one retained the drainage tube until the end of follow-up, and five underwent drainage tube removal; the mean drainage tube removal time was 149.17 ± 134.19 days. The efficacy of TACE leading to liver abscess was evaluated as partial response (18.75%), stable disease (37.5%), and progressive disease (43.75%). Eleven patients died during the follow-up period owing to causes unrelated to infections caused by liver abscesses. The survival rates at 3 months, 6 months, 1 year, and 5 years were 86.7%, 50.9%, 25.5%, and 17%, respectively.

**Conclusion:**

Patients with liver abscess formation following TACE for malignant liver tumors experienced prolonged drainage tube removal time after PCD; while this condition did not directly cause death, it indirectly contributed to a poor prognosis in these patients.

## Introduction

Transarterial chemoembolization (TACE) is an effective method for the treatment of unresectable malignant liver tumors (1, 2). With the development of embolization concepts and advances in embolization materials, TACE has become the preferred non-surgical treatment for unresectable primary liver cancer and liver metastases (3). It can embolize multiple lesions in a single course of treatment and can be applied repeatedly to the same patient (4).

However, TACE has a series of complications, including post-embolization syndrome (PES), hepatic impairment, and leukopenia, which are common and mild (5). In contrast, a liver abscess is a rare and serious complication. Its incidence is approximately 0.22%–4.46% (5, 6), and it may prolong hospital stay, delay tumor treatment, and even lead to death from severe infection (7–9). The risk factors for liver abscess after TACE include diabetes, biliary abnormalities, large tumor size, and portal vein occlusion (10–13).

Antibiotics combined with percutaneous catheter drainage (PCD) is the mainstream method for the treatment of liver abscess, but there are only a few studies on the therapeutic effect of liver abscess after TACE (8, 9, 12, 13). In addition, the relationship between TACE-induced liver abscess formation and tumor prognosis remains poorly understood. Abscess formation may promote tumor necrosis, but its inflammatory microenvironment may trigger tumor proliferation, migration, and other malignant processes.

Thus, this study aimed to retrospectively examine the outcomes of patients who developed liver abscesses following TACE to elucidate the impact of liver abscess formation on the prognosis of these patients.

## Methods

### Patients

This study was approved by the ethics committee of our hospital and complies with the principles of the Declaration of Helsinki. Owing to the retrospective nature of this study, the informed consent requirement was waived. The data were anonymized to protect patient privacy. Data were extracted from electronic medical records of patients with malignant liver tumors treated with TACE between January 2017 and January 2022. The characteristics of patients with complicated liver abscesses were reviewed.

The variables of interest included sex, age, comorbidities (e.g., diabetes), tumor status (primary/secondary), tumor diameter, liver function (Child–Pugh class), major surgical history, TACE status (session count, embolic materials used, and session duration), and liver abscess status (clinical symptoms, diagnosis time, and treatment method).

Follow-up assessments included the following variables: liver abscess healed, tumor response, drainage tube removal time, and survival status.

### Diagnosis of liver abscess

The diagnostic criteria for liver abscess included contrast-enhanced computed tomography (CT) images showing one or more focal hypodense lesions in the liver that may contain gas and the observation of persistent fever or chills. In addition, one of the following two conditions had to be met: a) blood culture was positive for bacteria; b) aspirated fluid contained a typical purulent substance or was positive for pus culture.

### Treatment of liver abscess

In our clinical practice, broad-spectrum antibiotics were applied to patients with suspected liver abscesses after TACE (14). If the infection was not effectively controlled after simple antibiotic treatment, PCD was performed. Infection control was defined as the disappearance of associated clinical symptoms and infection-related indicators returned to normal. The criteria for post-PCD tube removal included the absence of clinical symptoms and a drainage volume of <10 mL/day for three consecutive days and the follow-up CT scan findings showing no evidence of the abscess or <2 cm in size.

### Follow-up

All patients with liver abscess formation after TACE were followed up. All patients underwent contrast-enhanced CT/magnetic resonance imaging (MRI) scanning 10–60 days after liver abscess formation to determine liver abscess healed and tumor response. Subsequently, patients were followed up every 2–3 months. Patients who could not visit the hospital were followed up via telephone. Survival was the outcome of interest. If patients developed a second liver abscess, it was recorded as a follow-up endpoint for the first liver abscess. The deadline for the follow-up was December 31, 2022.

Liver abscess healed was defined as infection under control, which no longer needed PCD or intravenous antibiotics. Tumor response represented the therapeutic efficacy of TACE leading to liver abscess formation, which was determined based on contrast-enhanced CT/MRI findings within 1–3 months after TACE using the modified Response Evaluation Criteria in Solid Tumors (15).

### Statistical analyses

Data were expressed as means ± standard deviations or percentages. Statistical analysis was performed using SPSS 26.0 (IBM SPSS, Armonk, NY, USA).

## Results

**Figure 1:**
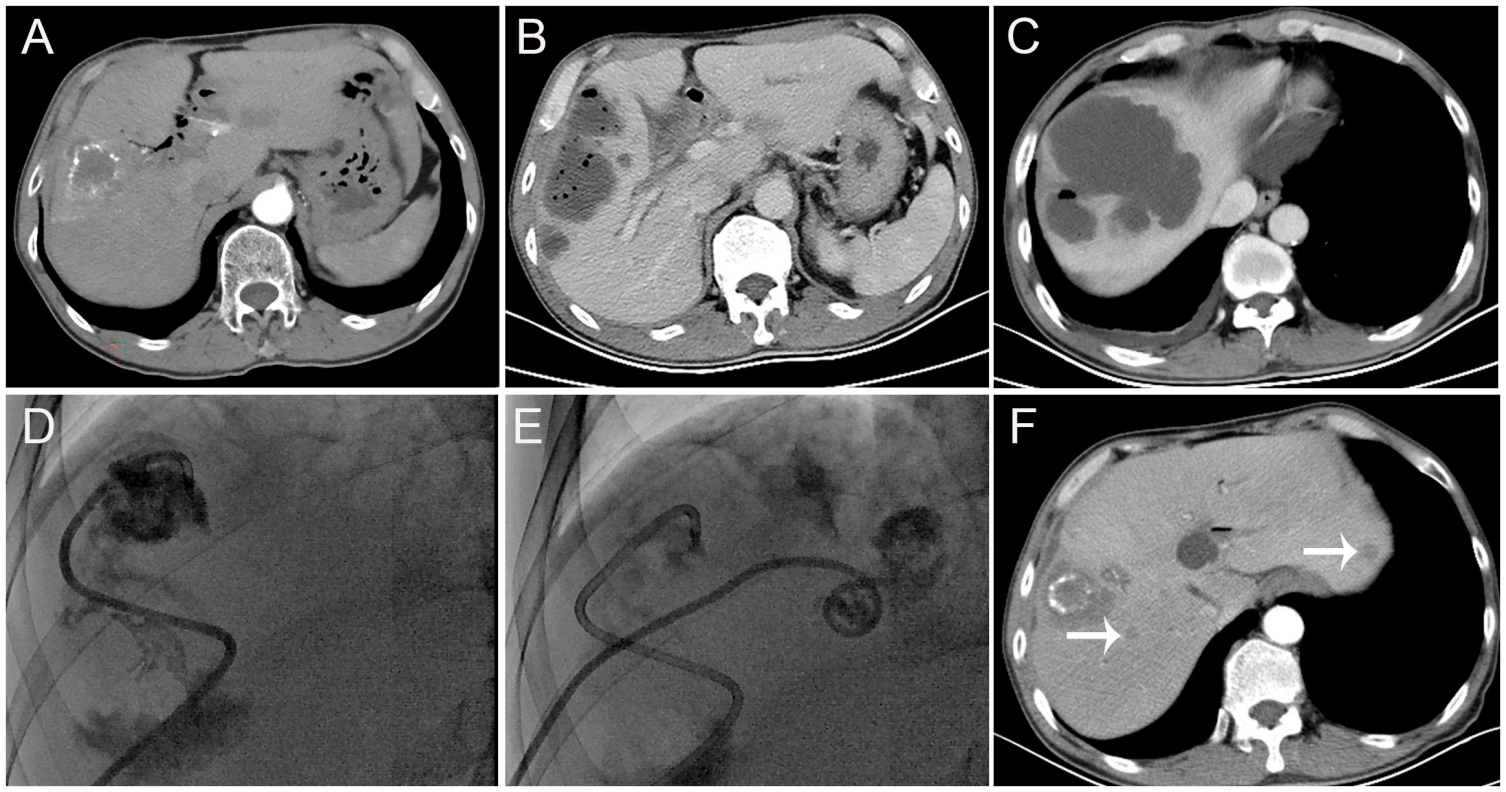
Findings from a 62-year-old patient with liver metastasis of cholangiocarcinoma and a history of biliary-enteric anastomosis and one previous transarterial chemoembolization (TACE) session. **(A)** Before the TACE session, contrast-enhanced computed tomography scans revealed hypodense lesions in segment V of the liver, with no significant enhancement. A small amount of iodized-oil emulsion deposit was observed, and the intrahepatic bile duct showed multiple pneumobilia and dilatations. **(B, C)** Thirteen days after this TACE session, contrast-enhanced computed tomography scans revealed the formation of multiple gas-containing liver abscesses. **(D, E)** Subsequently, the patient underwent percutaneous drainage (PCD). **(F)** One month after PCD, contrast-enhanced computed tomography scans showed an increase in tumor size and multiple new metastases in the liver (arrow).

A total of 1,387 patients with malignant liver tumors underwent 3,341 TACE sessions at our hospital between January 2017 and January 2022. Among them, 15 (1.1%) cases were complicated with liver abscess, resulting in a total of 16 (0.5%) liver abscess cases after 3,341 TACE sessions (including one patient with two events) (**Figures 1A–C**). The patients’ baseline characteristics are presented in **Table 1**.

**Table 1:** Baseline characteristics of patients with liver abscesses.

Patient	Age/sex	DM	Diagnosis	Surgical history	Portal vein thrombosis	Child–Pugh class
1	64/M	–	HCC	Pulmonary lobectomy	–	A
2	69/M	–	Liver metastasis of CCA	Whipple surgery	–	A
3	69/M	–	HCC	Partial hepatectomy RFA Biliary incision for stone removal	–	B
4.1	47/M	–	Liver metastasis of SPN	Whipple surgery	+	A
4.2	49/M	–	Liver metastasis of SPN	Whipple surgery	+	B
5	74/M	+	HCC	RFA	+	A
6	79/F	–	HCC	Meningioma resection	+	A
7	65/M	–	HCC	Partial hepatectomy RFA	–	A
8	62/M	–	Liver metastasis of CCA	Whipple surgery	–	A
9	56/F	–	HCC	Partial hepatectomy	–	A
10	65/M	–	Liver metastasis of VPC	PTCD	–	A
11	61/F	–	Liver metastasis of GIST	GIST and meningioma resection	–	A
12	54/M	–	Liver metastasis of CCA	Whipple surgery	–	A
13	64/F	+	HCC	PTCD	–	B
14	78/M	+	HCC	–	–	A
15	64/M	+	HCC	PTCD	–	A

Patients 4.1 and 4.2 refer to the same patient who presented twice with liver abscesses. The total number of patients was 15, and the total number of TACE cases was 16.

CCA, cholangiocarcinoma; DM, diabetes mellitus; F, female; GIST, gastrointestinal stromal tumors; HCC, hepatocellular carcinoma; M, male; PTCD, percutaneous transhepatic cholangial drainage; RFA, radiofrequency ablation; SPN, solid pseudopapillary neoplasm; TACE, transarterial chemoembolization.

Eight (50%) TACE sessions were “first” sessions; the mean number of sessions received was 2.31 ± 2.2 (range, 1–9). The mean maximum tumor diameter was 7.09 ± 2.86 (range, 2.8–12.4) cm. Five, one, and 10 TACE sessions were performed using iodized-oil emulsion (IOE), IOE and gelatin sponge particles, and drug-eluting beads (100–300 μm), respectively. The mean TACE duration was 109.06 ± 37.87 (range, 60–180) min. The mean interval from TACE to the diagnosis of liver abscess formation was 14.19 ± 9.21 (range, 3–40) days. The most common symptom was fever (93.8%), followed by chills (43.8%) and abdominal pain (31.3%). One patient received antibiotics only, while 14 patients received PCD combined with antibiotic treatment (**Figures 1D, E**). The infection caused by liver abscesses was effectively controlled in all patients after treatment. *Enterococcus faecalis* (31.25%) was the most common bacterial species detected in blood/pus cultures (**Table 2**).

**Table 2:** Clinical characteristics and surgical management of patients with liver abscesses.

Patient	TACE sessions	Treated tumor diameter (cm)	Embolic materials	TACE duration (min)	Symptoms	Diagnosis time (day)	Management	Blood or pus culture
1	1	12.3	IOE+GSP	60	Fever, chills, abdominal pain	30	AT	–
2	1	5.3	DEB	150	Fever, chills, abdominal pain	17	AT+PCD	*E. faecalis*
3	2	5.6	IOE	105	Abdominal pain	40	AT+PCD	*E. coli*
4.1	2	12.4	IOE	90	Fever	23	AT+PCD	Negative
4.2	9	5.9	IOE	120	Fever	6	AT+PCD	Negative
5	6	8.0	IOE	90	Fever	15	AT+PCD	Negative
6	2	4.9	DEB	180	Fever, chills	10	AT+PCD	*E. coli*; *E. faecalis*
7	1	2.8	IOE	90	Fever, chills	3	AT+PCD	*K. pneumonia*
8	2	5.0	DEB	120	Fever	13	AT+PCD	*E. faecalis*
9	1	7.4	DEB	180	Fever, chills	11	AT+PCD	*E. coli*
10	2	9.4	DEB	90	Fever, abdominal pain	15	AT+PCD	*E. faecalis*
11	1	9.7	DEB	90	Fever	10	AT+PCD	Negative
12	1	3.5	DEB	60	Fever	12	AT+PCD	*E. coli*; *M. morgan*
13	1	5.0	DEB	150	Fever, chills	4	AT+PCD	Negative
14	1	8.6	DEB	90	Fever	3	AT+PCD	Negative
15	4	7.6	DEB	80	Fever, chills, abdominal pain	15	AT+PCD	*E. faecalis*

AT, therapy; Diagnosis time, time from TACE to the discovery of liver abscess; DEB, drug-eluting beads; E. coli, Escherichia coli; E. faecalis, Enterococcus faecalis; GSP, gelatin sponge particles; IOE, iodized-oil emulsion; K. pneumonia, Klebsiella pneumonia; M. morgan, Morganella morgan; PCD, percutaneous drainage; TACE, transarterial chemoembolization.

Follow-up assessments after PCD revealed six (40%) abscess cases were healed, nine (60%) abscess cases did not heal completely, eight patients died before drainage tube removal, one patient retained the drainage tube until the end of the follow-up, and five patients underwent drainage tube removal. The mean drainage tube removal time was 149.17 ± 134.19 (range, 73–420) days. A total of zero, three (18.75%), six (37.5%), and seven (43.75%) cases showed complete response (CR), partial response, stable disease, and progressive disease (PD), respectively (**Figure 1F**). Eleven patients died during the follow-up period owing to causes unrelated to infections caused by liver abscesses (**Table 3**). The survival rates at 3 months, 6 months, 1 year, and 5 years were 86.7%, 50.9%, 25.5%, and 17%, respectively.

**Table 3:** Treatment and follow-up findings in patients with liver abscesses.

Patient	Treatment	Infection controlled	Follow-up time (months)	Abscess healed	Tumor response	Drainage tube removed	Drainage tube removal time (days)	Survival status
1	AB	+	18	+	SD	NA	NA	Survival
2	AB+PCD	+	5	+	SD	+	73	Death
3	AB+PCD	+	4	−	PD	−	NA	Survival
4.1	AB+PCD	+	49	+	PR	+	75	Survival
4.2	AB+PCD	+	23	+	PD	+	420	Survival
5	AB+PCD	+	7	−	PR	−	NA	Death
6	AB+PCD	+	10	+	PD	+	120	Death
7	AB+PCD	+	3	−	SD	−	NA	Death
8	AB+PCD	+	6	−	PD	−	NA	Death
9	AB+PCD	+	4	−	PR	−	NA	Death
10	AB+PCD	+	5	−	SD	−	NA	Death
11	AB+PCD	+	18	−	SD	−	NA	Death
12	AB+PCD	+	9	+	PD	+	90	Death
13	AB+PCD	+	2	−	SD	−	NA	Death
14	AB+PCD	+	6	+	PD	+	117	Death
15	AB+PCD	+	9	−	PD	−	NA	Death

Tumor response was the response observed after the confirmation of liver abscess by contrast-enhanced computed tomography/magnetic resonance imaging scanning within 1–3 months after the formation of liver abscess. The mRECIST criteria were used as the evaluation standard. AB, antibiotic; CR, complete response; NA, not applicable; PCD, percutaneous drainage; PD, progressive disease; PR, partial response; SD, stable disease; mRECIST, modified Response Evaluation Criteria in Solid Tumors.

## Discussion

PCD is an effective treatment for liver abscess formation after TACE to control infection (13, 16); however, our study showed that its cure rate was low, mainly due to the inability to remove the drainage tube. In addition, the drainage tube removal time in this study was longer than that in patients with community-acquired liver abscesses (17, 18). Delayed or failed extubation may be the result of the connection between abscesses and bile ducts or even biloma formation (19).

One study has argued that complete liquefactive necrosis of tumor lesions may be indicative of a good prognosis and that the discharge of necrotic tumor tissue fluid via the drainage tube may have positive implications for tumor treatment (13). However, the findings of our study were not consistent with previous findings. In this study, no cases were evaluated as CR, while cases with PD accounted for 43.75%. Furthermore, 11 patients died during the follow-up period. The formation of a liver abscess delayed subsequent treatment and was bound to affect the prognosis, although the infection caused by the abscess could be controlled. Liver abscess was not directly related to death, but it led indirectly to poor prognosis in these patients.

History of biliary-enteric anastomosis is an important risk factor for liver abscess formation (6, 9, 10). In this study, 53.3% of the patients had a history of biliary surgery. Some studies have found that Oddi sphincter dysfunction or incision permitted retrograde intestinal bacteria entry into and colonization of the bile duct (8, 20). Meanwhile, the local toxicity of chemoembolic agents and the embolization of bile duct feeding vessels resulting from TACE may lead to bile duct injury (21). In this study, patients had received an average of 2.31 ± 2.24 TACE sessions at the time of liver abscess formation; among them, one patient developed liver abscesses after the second and ninth TACE sessions. Bile duct injury following TACE may enable opportunistic pathogens to colonize the bile duct and enter the liver parenchyma, where they undergo rapid proliferation within the local ischemic and hypoxic microenvironment after TACE, thus leading to liver abscess formation (6, 22, 23).

Diabetes is also thought to be a predisposing factor for liver abscess formation after TACE.

Diabetes is also thought to be a predisposing factor for liver abscess formation after TACE (9, 10). On the one hand, the chronic inflammatory state that arises in diabetes leads to continuous reactive oxygen species (ROS) production, and ROS stimulate the expression of pro-inflammatory cytokines by activating transcription factors such as nuclear factor-kappa B (24). In addition, in diabetic patients, excess adipose tissue secretes pro-inflammatory cytokines, further amplifying oxidative stress (25). On the other hand, hyperglycemia in diabetes is thought to cause dysfunction of the immune response, which fails to control the spread of invading pathogens in diabetic subjects, through the following mechanisms: impairment of cytokine production, leukocyte recruitment inhibition, defects in pathogen recognition, neutrophil dysfunction, macrophage dysfunction, natural killer cell dysfunction, and inhibition of antibodies and complement effector (26).

In this study, 87.5% of the tumors had a maximum diameter of >5 cm. Large tumor size and extensive use of embolic materials may have simultaneously increased the extent of intrahepatic necrosis and the risk of abscess formation (9). In addition, portal vein tumor thrombosis and gelatin sponge use have been associated with liver abscess formation (8). Owing to the small sample size, we could not examine the association between these risk factors and liver abscess formation.

It has been suggested that patients with liver metastases are more likely to develop liver abscesses after TACE compared with hepatocellular carcinoma (HCC) (27). In HCC, high concentrations of chemoembolic agents in the tumor tissue after infusion can be achieved because the arteries that feed the tumor and intratumoral blood space are mostly dilated. On the contrary, the feeding artery and the intratumoral blood space of metastatic liver tumors are not usually dilated, thus decreasing the intratumoral concentration of chemoembolic agents. This may result in higher concentrations of the chemoembolic agents in the surrounding liver parenchyma and initiation of biliary epithelial damage. Additionally, HCC is mostly noted in cirrhotic livers that are known to have dilation of the perivascular plexus, which can serve as a porto-arterial shunt and compensate for the decreased arterial flow (27, 28). Therefore, they may be the reasons for the higher incidence of liver abscesses in patients with liver metastases.

Prevention of the occurrence of liver abscesses after TACE has been the focus of clinical attention. Incidentally, it is unreasonable and unnecessary to use antibiotics prophylactically for all patients with TACE. It has been shown that prophylactic antibiotics reduce the incidence of liver abscess formation in patients with bile duct injury (29, 30). Moreover, previous studies have shown that the incidence of liver abscess formation does not increase among patients without bile duct injury who do not receive prophylactic antibiotics (30, 31). Therefore, stratification according to risk factors is more reasonable for the administration of prophylactic antibiotics before TACE.

In this study, 15 of 1,387 (1.1%) patients undergoing TACE subsequently developed liver abscesses; this rate was similar to that previously reported (5–13, 16, 19, 27, 32–36) (**Table 4**). Although the incidence of liver abscess formation after TACE is low, it is necessary to grasp its clinical symptoms. In our study, the most common symptom reported by patients was fever (93.8%), followed by chills (43.8%) and abdominal pain (31.3%). However, PES characterized by fever, right upper quadrant pain, nausea, and vomiting is the most common adverse event, affecting approximately 60%–80% of patients. PES generally occurs immediately after TACE, with fever peaking within 2 days after TACE, and it is often self-limiting and does not require antibiotic treatment (37–40). In contrast, in this study, the mean interval from TACE to the discovery of liver abscess was 14.19 ± 9.21 (range, 3–40) days, suggesting that the onset of liver abscess formation occurred later than PES at the time point after TACE. Furthermore, chills are frequently observed among patients with liver abscesses but not in those with PES, which may help differentiate between these outcomes.

**Table 4:** Review of literature on the incidence of liver abscess after TACE.

Study	Year	Place	Study design	Setting	No. of patients who underwent TACE	No. of patients with liver abscesses after TACE	Incidence of liver abscess after TACE
Zhu et al.	2022	China, mainland	Retrospective	2 hospitals	11,524	84	0.72%
Arslan et al.	2019	Turkey	Retrospective	1 hospital	163	4	2.45%
Jia et al.	2018	China, mainland	Retrospective	4 hospitals	3,129	23	0.74%
Sun et al.	2017	China, mainland	Retrospective	1 hospital	1,480	5	0.34%
Lv et al.	2016	China, mainland	Retrospective	1 hospital	3,613	21	0.58%
Shin et al.	2014	South, Korea	Retrospective	1 hospital	5,299	72	1.36%
Xia et al.	2006	China, mainland	Retrospective	1 hospital	1,348	3	0.22%
Huang et al.	2003	China, Taiwan	Retrospective	1 hospital	1,347	7	0.52%
Kim et al.	2001	United States	Retrospective	1 hospital	157	7	4.46%
Song et al.	2001	South, Korea	Retrospective	1 hospital	2,439	14	0.57%
Tarazov et al.	2000	Russia	Retrospective	1 hospital	282	6	2.13%
Gates et al.	1999	United States	Retrospective	1 hospital	251	1	0.40%
Sakamoto et al.	1998	Japan	Retrospective	1 hospital	850	5	0.59%
Chen et al.	1997	China, Taiwan	Retrospective	1 hospital	289	5	1.73%
de Baère et al.	1996	France	Retrospective	1 hospital	181	3	1.66%
Farinati et al.	1996	Italy	Retrospective	1 hospital	72	2	2.78%
Reed et al.	1994	United States	Retrospective	1 hospital	227	6	2.64%

TACE, transarterial chemoembolization.

This study has some limitations. First, as this was a single-center study, the results cannot be generalized. Second, as this was a retrospective study, our data integrity may not be as high as that in prospective studies or clinical trials. Third, as some patients after TACE were subsequently lost to follow-up, this study may have underestimated the incidence of liver abscess formation following TACE. Fourth, the low incidence of post-TACE liver abscess and small sample size precluded comparative analyses of prognosis in patients without liver abscess formation. Moreover, we could not use univariate and multivariate Cox regression statistical methods to reach more robust conclusions.

In conclusion, patients with liver abscess formation following TACE for malignant liver tumors had a long drainage tube removal time after PCD and even required continuous canalization. Although liver abscess formation was not directly related to death, it indirectly led to poor prognosis in these patients.

## Data availability statement

The original contributions presented in the study are included in the article/supplementary material. Further inquiries can be directed to the corresponding author.

## Ethics statement

This study protocol was reviewed and approved by the Ethics Committee of Shengjing Hospital of China Medical University (protocol number 2022PS1175K). The studies were conducted in accordance with the local legislation and institutional requirements. Owing to the retrospective nature of this study, the informed consent requirement was waived.

## Author contributions

YW: Writing – original draft. ZC: Data curation, Funding acquisition, Writing – review & editing. JHZ: Formal Analysis, Writing – review & editing. ZL: Data curation, Writing – original draft. JZ: Writing – review & editing.
